# Extra-Neural Metastases of Glioblastoma: A Case Series

**DOI:** 10.7759/cureus.47556

**Published:** 2023-10-24

**Authors:** Catarina Almeida, Marta Baptista Freitas, Andreia Costa, Catarina Fernandes, Miguel Barbosa

**Affiliations:** 1 Medical Oncology, Centro Hospitalar Universitário de São João, Porto, PRT

**Keywords:** radiotherapy (rt), temozolomide, extraneural metastases, stupp protocol, glioblastoma

## Abstract

Glioblastoma (GBM) is the most common brain tumor and has a median survival of less than two years. The current standard of care consists of surgery followed by concomitant radio and chemotherapy with temozolomide. Although it is an aggressive tumor, distant metastases are extremely rare. The dissemination mechanisms are not fully understood and currently there is no standard of care for its treatment. In this study, the authors present a comprehensive analysis of all the existing cases of extra-neural metastases of GBM in a tertiary care center over the last five years, along with the procedures carried out in each case.

## Introduction

Glioblastoma (GBM) is the most common malignant brain tumor, making up 46.1% of all primary brain tumors [1]. This type of tumor is slightly more frequent in males, with an incidence 1.6 times higher than in females and its incidence tends to increase with age [2]. It has the worst prognosis among all brain tumors due to its anaplasia, high mitotic activity, microvascular proliferation, and necrosis [3]. This has resulted in the assignment of the highest grade (grade 4) of the World Health Organization (WHO) classification of brain tumors. Its aggressiveness and infiltration are the reasons why GBM tends to relapse and quickly evolve regardless of the recommended treatment, resulting in high mortality. Despite its local aggressiveness, this type of tumor does not usually have systemic dissemination, and extra-neural metastases are considered extremely rare, occurring in only 0.2-0.4% of cases [4]. Within its rarity, the most common sites of metastases are lymph nodes, bones, lungs, and liver [4,5]. There is currently no standard of care for metastatic glioblastoma and the treatment is thoroughly deliberated considering each patient’s history of disease, metastases location, and general status.

This study aimed to report all cases of extra-neural metastases of GBM that were diagnosed in the University Hospital Center of São João since 2017 in order to increase physicians' awareness of this rare neoplasm and its treatment approaches in clinical practice.

## Case presentation

From January 2017 to December 2021, 314 cases of GBM were diagnosed in adults in our center, three of which had extra-neural metastases (0.95%).

Case 1

**Figure 1:**
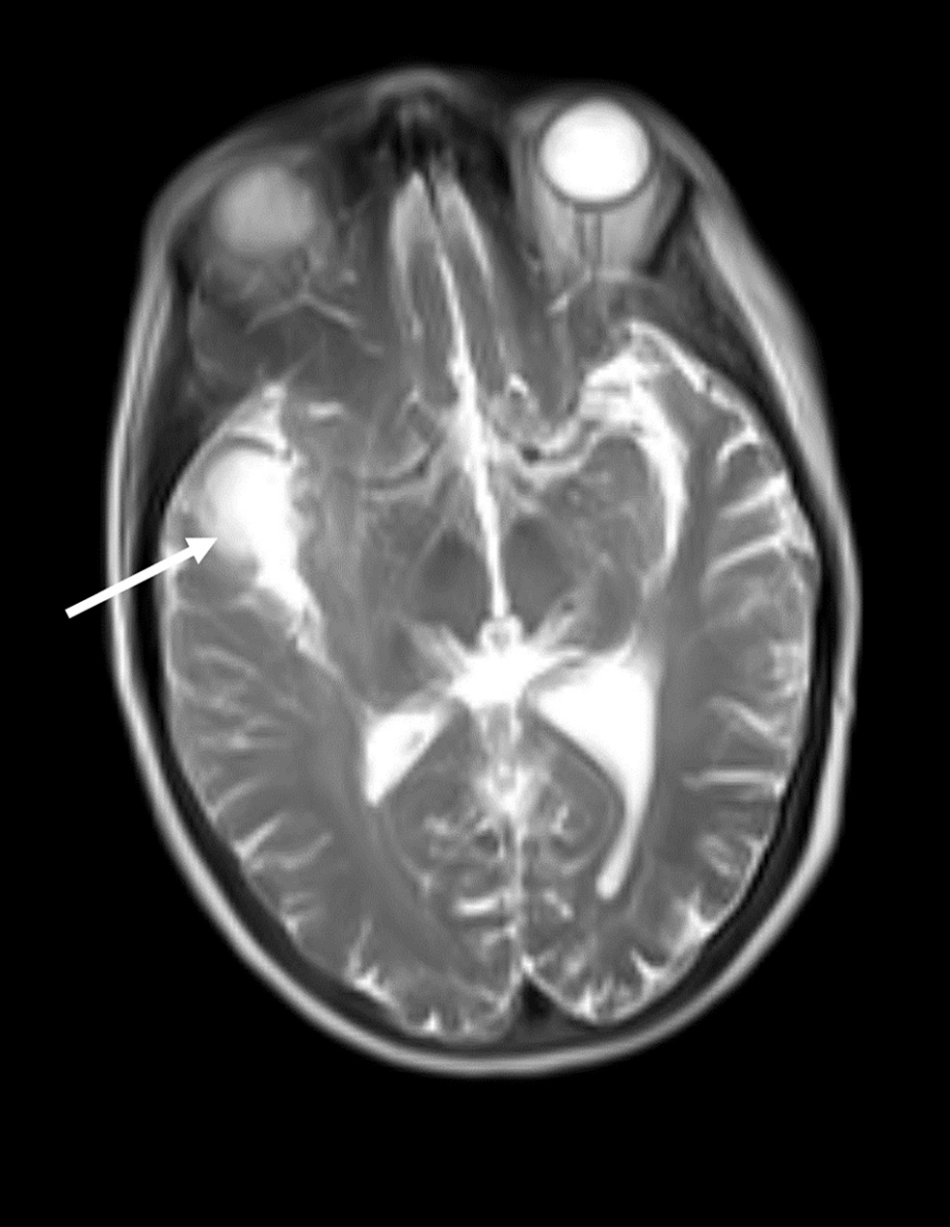
MRI image showing an expansive lesion in the right frontal region measuring 58 × 38 × 50 mm.

A 75-year-old female presented with sudden decreased muscle strength in the left arm. She resorted to the emergency service and a head computed tomography (CT) was immediately made, describing a lesion compatible with a malignant tumor. A cerebral magnetic resonance imaging (MRI) was performed to clarify these findings and it showed a large expansive lesion in the right frontal region measuring 58 × 38 × 50 mm, which can be seen in Figure 1. It was described as being highly suspicious of a high-grade glioma.

The tumor was subjected to complete resection of the lesion and the anatomopathological (AP) report revealed a GBM isocitrate dehydrogenase (IDH) wildtype without methylguanine methyltransferase (MGMT) gene promoter methylation. She was treated with concomitant radiotherapy (RT) and chemotherapy according to the modified Stupp regimen (36 Gy administered in 2.4 Gy fractions over three weeks with concomitant temozolomide 75 mg/m^2^, followed by temozolomide 150 mg/m^2^ for five days every 28 days) [6]. Three months after the surgery, while doing the first cycle of adjuvant temozolomide, the patient presented a worsening state of consciousness with a slow mental response, memory loss, and inability to walk, becoming bedridden. However, the MRI did not show signs of disease progression. Around the same time, the patient was diagnosed with a pathological subtrochanteric fracture on the left hip and underwent surgery in a different hospital. The histology report of the bone was compatible with GBM metastasis. Due to the worsening state of consciousness, it was decided to suspend treatment and maintain follow-up by the palliative care team of her residence area; the patient was lost to follow-up.

Case 2

**Figure 2:**
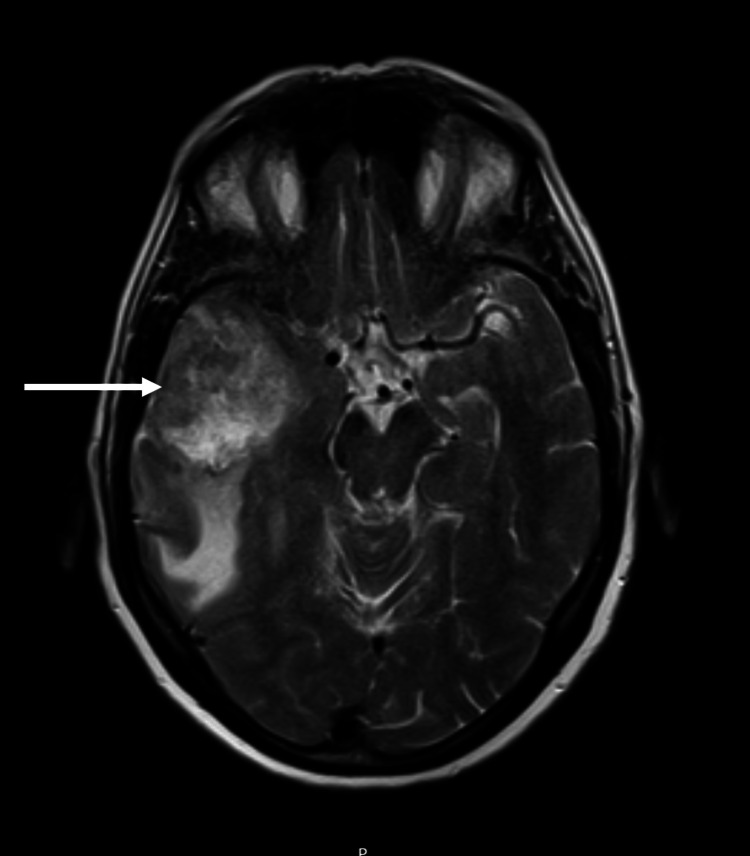
MRI showing a right frontal temporal lesion measuring 43 × 38 × 43.5 mm.

A 61-year-old female had headaches, vomiting, and decreased muscle strength in the upper and lower left limbs as presenting symptoms. After the head CT showed a space-occupying injury, the cranial MRI displayed a right frontal temporal lesion measuring 43 × 38 × 43.5 mm suggestive of high-grade glioma, as shown in Figure 2.

The patient was submitted to surgery and the post-operative MRI showed tumor residue, therefore it was considered an incomplete resection; the AP report indicated that it was an IDH-wildtype GBM. Consequently, the patient initiated the Stupp protocol (60 Gy administered in 2.0 Gy fractions over six weeks with concomitant temozolomide 75 mg/m^2^, followed by temozolomide 150 mg/m^2^ for five days every 28 days) [6].

**Figure 3:**
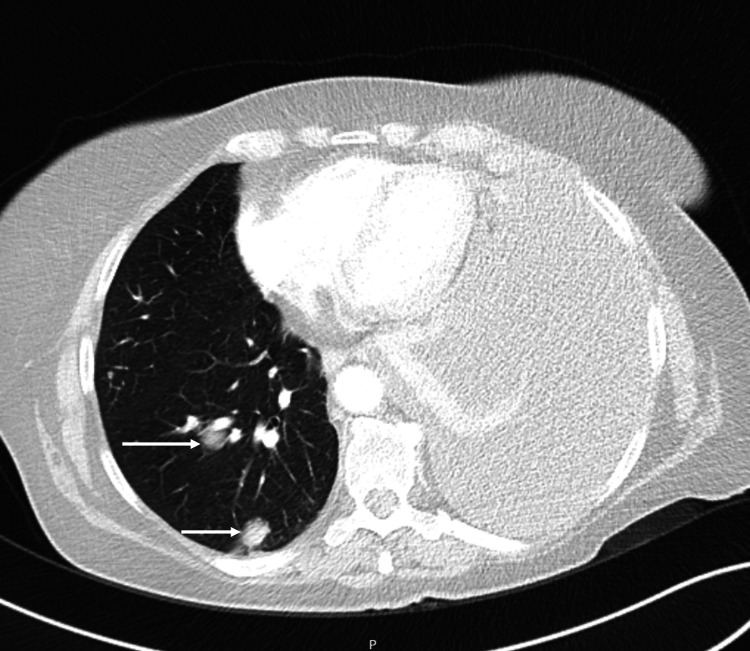
Pulmonary metastatic nodules.

**Figure 4:**
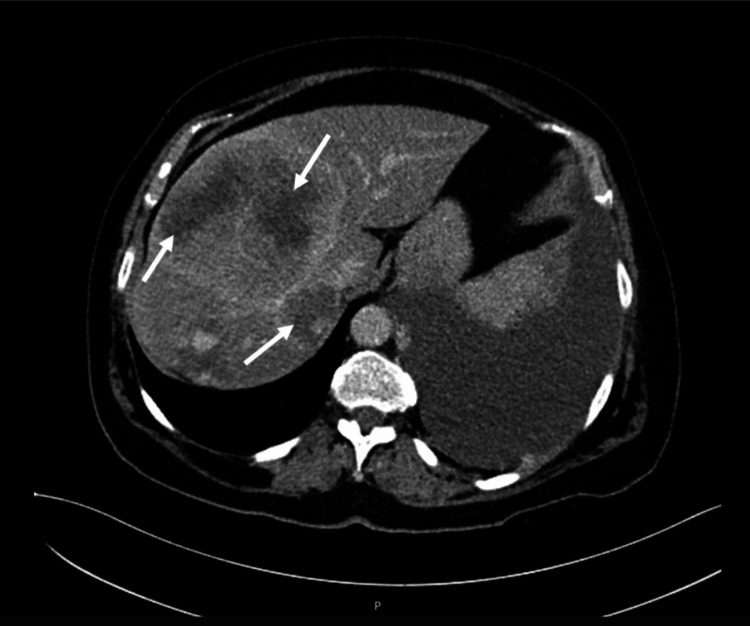
Abdominal CT scan with liver nodules suggestive of metastases.

After the seventh cycle of adjuvant temozolomide, the patient was admitted to the hospital due to large volume pleural effusion causing dyspnea and heavy coughing. The patient was admitted to the inpatient clinic and the physicians proceeded with the investigation of what was thought to be another primary tumor. In the etiological study that was carried out, the CT highlighted the thickening of the pleural leaflets, multiple metastatic pulmonary nodules, and four hepatic nodules (Figures 3, 4). 

**Figure 5:**
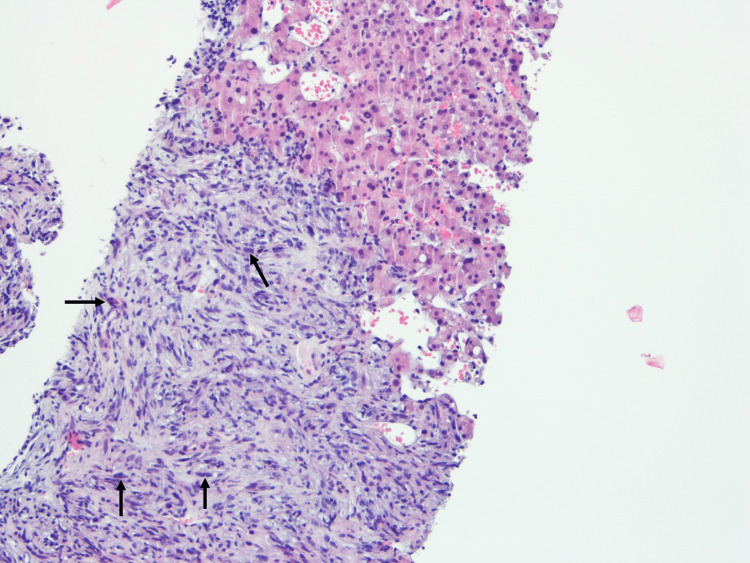
Histopathology of hepatic nodule supporting the diagnosis of GBM metastasis (H&E, 100×). GBM: glioblastoma

The pleural fluid anatomopathology was negative for malignant cells and no alterations were found in the mammography, thyroid ultrasound, endoscopy, and colonoscopy. The liver biopsy, scheduled for later, revealed GBM metastasis (Figure 5). Regarding the anatomopathological findings, we can observe regions that are more densely cellular, with greater nuclear pleomorphism and several mitotic figures.

During hospitalization the patient had cognitive and neurological deterioration, thus it was decided for best supportive care strategy; given that the patient was not submitted to any other invasive techniques, the pulmonary metastases were never confirmed. She passed away seven days after confirmation of metastasis.

Case 3

**Figure 6:**
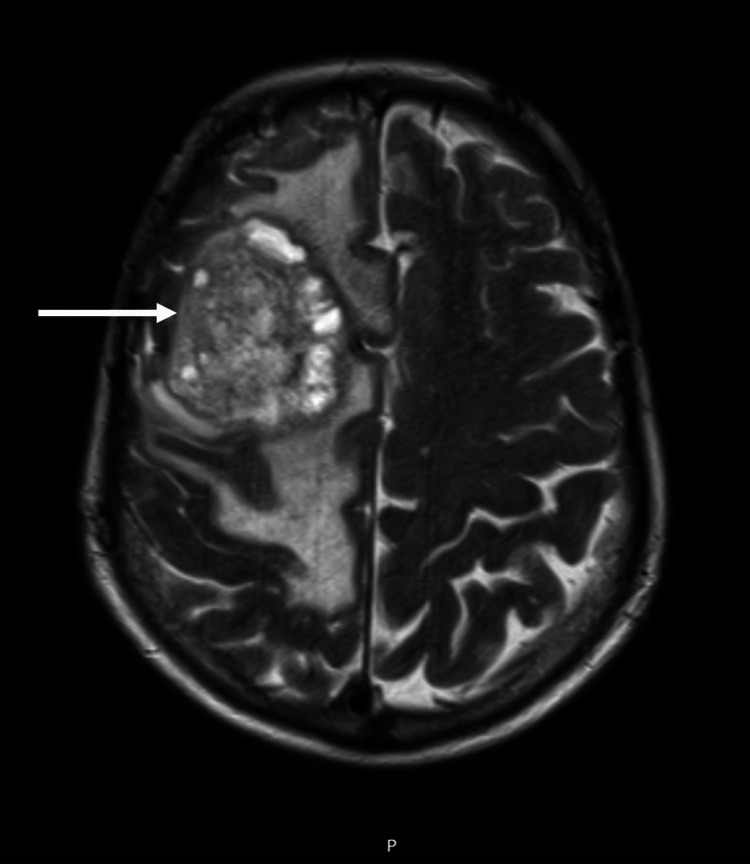
MRI showing a right frontal lesion of 4 cm in the right frontal lobe.

A 64-year-old male had gradual muscle strength loss in the upper and lower left limbs and left central facial paresis for roughly six weeks. The first examination that was made was a head CT, and based on the findings, he was subjected to an MRI, which showed a right frontal lesion of 4 cm, highly suspicious of a high-grade glioma (Figure 6).

**Figure 7:**
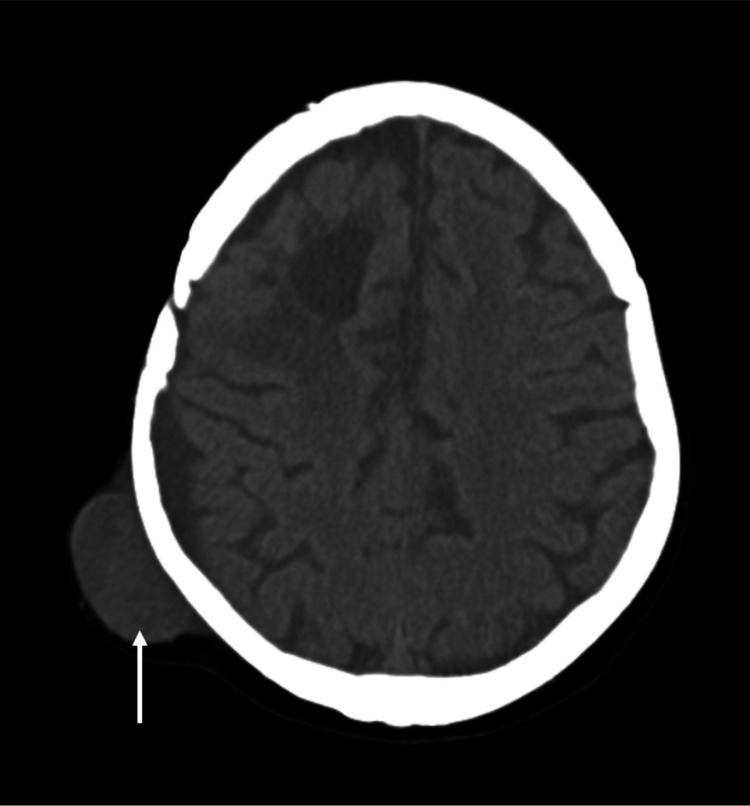
Brain CT with right parietal epicranial expansive lesion.

**Figure 8:**
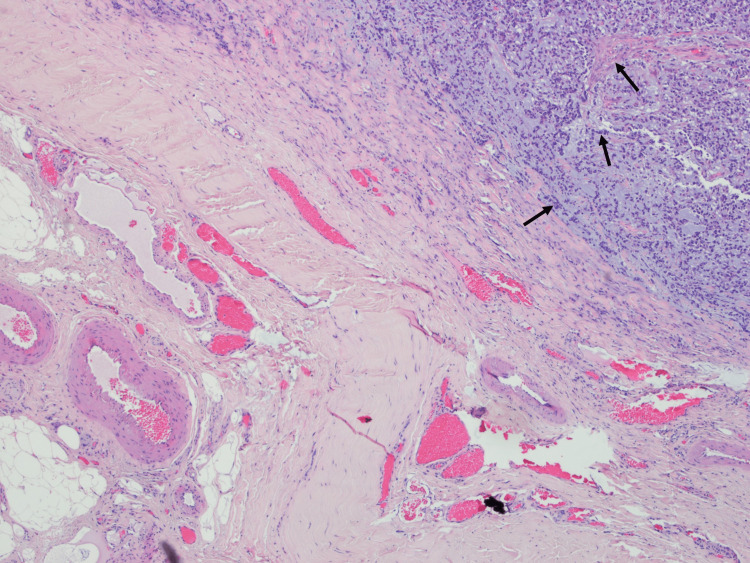
Histological findings of excisional biopsy of subcutaneous lesion, supporting the diagnosis of GBM metastasis (H&E, 100×). GBM: glioblastoma

He underwent complete macroscopic resection of the lesion and the anatomopathological report revealed an IDH-wildtype GBM. The patient recovered from focal deficits after surgery and initiated treatment with Stupp protocol. After the first cycle of adjuvant temozolomide the patient had two generalized tonic-clonic seizures and the MRI displayed signs of disease progression. Therefore, he initiated second-line chemotherapy with lomustine and bevacizumab approximately seven months after the surgery. After two cycles, swelling in the posterior region of the craniotomy was clinically observed. The cerebral MRI presented an expansive right parietal epicranial lesion (Figure 7), whose excisional biopsy confirmed GBM dissemination (Figure 8).

There was no evidence of progression regarding the primary cerebral lesion. Therefore, the patient resumed treatment with bevacizumab and lomustine and started external RT to the scalp with palliative intent, with a total dose of 20 Gy, at 4 Gy per day, in five fractions, with 9 MeV electron energy. He died five months later, with disease progression.

## Discussion

Despite the medical advances regarding multimodality treatments with chemotherapy and RT, GBM bears an overall poor prognosis, with a median overall survival of 15 months with optimal treatment [7]. In addition to this, the natural course of disease bestows on patients many changes in their general condition, such as physical impairment and cognitive and neurological deterioration, taking away quality of life and overburdening the caregivers. The five-year overall survival rate is 5.4%, one of the lowest concerning all types of malignant tumors [8]. There is no standard of care in case of disease progression after treatment according to Stupp protocol; second-line treatment usually goes through chemotherapy with lomustine or irinotecan with the addition of bevacizumab [9]. Given the scarcity of GBM metastases, there are no trials regarding its treatment, only a few sporadic case reports, namely with metastases in lymph nodes, bones, lungs, liver, skin, and spleen [4-5,10-12]. In the great majority of the described cases, the patients have such an accelerated neurological decline that it was decided not to proceed with systemic treatment, but to go with the best supportive care instead. In some cases the therapeutic options range from exclusive RT, exclusive surgery, or just chemotherapy with temozolomide, nitrosoureas bevacizumab; or even a combination of these options [13].

There have been more documented cases of extra-neural metastases of GBM in the last few years. However, this number remains low due to the reduced survival rate, only some cases have time to disseminate. Furthermore, some of the described case reports were post-mortem findings, not displaying any signs or symptoms during lifespan [10-12,14]. This shows that probably there are many cases that remain undiagnosed since autopsies in cases of known cancer (mostly an aggressive type such as GBM) are a rare procedure.

Regarding the anatomopathological findings which characterize GBM, there are more densely cellular regions, with greater nuclear pleomorphism, several mitotic figures, necrosis, and vascular and endothelial cell proliferation [15].

The pathophysiological mechanisms of the systemic dissemination of GBM are still not completely clarified. One of which is the breaking of the blood-brain barrier, either by the tumor itself or the surgical procedure, permitting access to the blood and lymphatic vessels. The subcutaneous metastasis of the third case could be justified by the surgical approach, given the location. This mechanism appears to be related to a dissemination process and not exactly by distant metastasis. Other described mechanisms are the invasiveness of the tumor, resistance to the immunologic defense mechanisms, and adaptation to hostile environments through quiescence [13]. All of these mechanisms could explain the development of distant metastases in the two other cases. Advances and efforts have been made in exploring novel approaches such as immunotherapy and precision oncology [16]. The investigation and knowledge of this patient population may serve as a basis for developing the appropriate therapy for extra-neural metastatic GBM, for which there is no established treatment.

## Conclusions

The proportion of cases in this hospital and the survival rates after the diagnosis of metastases are congruent with existing literature, with the exception of the last case, whose mechanism seems to have been by dissemination after the surgical procedure and not distant metastasis. The diagnosis of extra-neural metastization of GBM provides a dismal prognosis, for which there is still no standardized treatment. The pathophysiological mechanisms of this systemic dissemination are quite unclear and may be useful in the development of appropriate therapy. This, along with the early referral to palliative care will bring not only more adequate treatment options and increase survival outcomes but also improve the quality of life of both patients and caregivers.
